# The impact of exclusive enteral nutrition on the intestinal microbiota in inflammatory bowel disease

**DOI:** 10.3934/microbiol.2018.4.584

**Published:** 2018-07-20

**Authors:** Andrew S Day

**Affiliations:** Department of Paediatrics, University of Otago Christchurch, Christchurch, New Zealand

**Keywords:** Crohn disease, inflammatory bowel disease, therapy, nutrition, intestinal microbiota, inflammation, dysbiosis

## Abstract

It is increasingly clear that the intestinal microbiota plays key roles in the pathogenesis of the conditions known as Crohn disease and ulcerative colitis (jointly known as the inflammatory bowel diseases). Perturbations of the microbiota, termed dysbiosis, are present at diagnosis and likely reflect earlier environmental influences along with interactions with intestinal immune responses. Over the last two decades, there has been increasing interest in the use of a nutritional therapy to induce remission of active Crohn disease. Amongst a number of recent studies focusing on the putative mechanisms of action of enteral nutrition in Crohn disease, there have been several reports illustrating profound interactions between this nutritional therapy and the intestinal microbiota. Although at present it is still not clear how these changes relate to concurrent improvements in inflammation, it has become an area of increasing interest. This review article focuses on the impacts of nutritional therapy in individuals with active Crohn disease and overviews the most recent data arising from international studies.

## 1. Introduction

The inflammatory bowel diseases (IBD) are characterised by chronic inflammatory changes involving the gastrointestinal tract (gut). Although the two main subtypes of IBD, Crohn disease (CD) and ulcerative colitis (UC), share similar features, CD is differentiated from UC by disease location, histological changes and disease behaviour [1]. For instance, CD can involve any section of the gastrointestinal tract, with areas of involvement interspersed with uninvolved areas (so-called skip lesions). On the other hand, UC typically involves continuous inflammation starting from the rectum and involving a variable length of the colon, without involvement of other areas of the gut.

The pathogenesis of IBD is still not completely understood. At present, the best accepted hypothesis is that environmental factors lead to key changes in the intestinal microbiota that then trigger dysregulated innate and acquired immune responses in individuals with genetic risk factors [2]–[4]. Important environmental factors include diet, antibiotic exposure, and enteric infections. More than 200 genes have now been linked with increased risk of IBD: some of these are specific to CD or UC while others (e.g. IL-23R and MUC19) relate to risk for both disease types.

At present IBD is incurable: management involves induction of remission followed by maintenance of remission [1]. Various treatments can be employed: these include medical therapies, nutritional interventions and surgery. Several of these interventions induce modulation of the intestinal microbiota. Nutritional therapies include the use of fully liquid diets to induce remission: termed exclusive enteral nutrition (EEN) [5],[6]. EEN has been clearly linked with changes in the intestinal microbiota. This review focuses specifically on the current understanding of the impact of EEN on the intestinal microbiota.

## 2. Exclusive enteral nutrition (EEN)

EEN involves the exclusive use of a liquid diet for a period of time (typically up to 8 weeks), with exclusion of normal diet over this time [5]. The potential role of EEN arose from descriptive studies more than four decades ago: these initial case reports and case series demonstrated improved inflammatory status in adult patients with CD managed with nutritional interventions [7]. A few years later, a randomised controlled study compared EEN and corticosteroids (CS) in Irish adults with active CD: in this study EEN and CS performed similarly [8]. Although numerous subsequent studies have shown benefits with EEN in adults, many more recent reports have shown that EEN is typically more efficacious in children than in adults [5],[6].

Although the main role of EEN is the induction of remission, this intervention also has various other benefits. These include enhancing nutritional status and improving bone health [5],[9],[10]. Furthermore, this intervention essentially has no side-effects and avoids the adverse effects seen with CS [1]. Refeeding syndrome, characterised by electrolyte abnormalities following the recommencement of nutritional intake after a period of relative or absolute starvation, has however been described in a handful of cases [11],[12].

Following a period of EEN, many units recommend ongoing maintenance enteral nutrition (MEN), involving supplementary enteral formulae intake supplied in addition to standard or habitual diet. This intervention is associated with maintenance of remission, with reduction in relapse [5],[13],[14]. Further, this strategy enhances nutrition and growth (especially in children). Recent Japanese studies also show that MEN enhances the benefits of biologic therapies (such as infliximab) and prevents relapse after surgically-induced remission [5],[15],[16].

## 3. The intestinal microbiota in the context of IBD

Many studies have focused on looking at a single organism as the causative factor for the development of IBD [17],[18]. Over time, putative candidates have included measles virus, *Campylobacter* species, *Mycobacterium paratuberculosis* (MAP) and *Escherichia coli* (e.g. adherent invasive *E. coli*). As one example, a number of studies illustrate higher rates of CD developing after Campylobacter infections. In addition, data shows a significantly greater prevalence of specific Campylobacter species (*C. concisus*) in children with CD compared to well control children [17]. Further, these organisms possess key virulence factors, are able to invade enterocytes and trigger local inflammatory responses. The occurrence of this organism is not, however, universal and nor does the carriage of this organism explain the full pattern of IBD. Overall, at present, there is not yet conclusive evidence that supports this or any other single microbe as the causative organism in the development of IBD.

Most recent data illustrate that the presence of distinct changes in the patterns of the flora, so called dysbiosis, are more important in the development of IBD. This dysbiosis is considered to be present prior to and at the onset of IBD and is characterised by variations in the balance between bacterial groups (e.g. Firmicutes and Bacteroidetes). Some recent data also indicate that a reduction in one particular group of bacteria, *Faecalibacterium prausnitzii* is paramount [19]. This bacterium possesses at least one protective protein with anti-inflammatory benefits [20]. A reduction in numbers of this organism is thought to then lead to altered innate defence responses, thereby raising the risk of developing IBD.

Several animal and human observations confer a critical role for the intestinal microbiota in the development of IBD. Firstly, the presence of microbiota is required to promote the development of inflammation in various animal models of IBD. An example is the interleukin (IL)-10 knock out mouse model [21]. Inflammation does not develop if these animals are housed in a germfree setting but does occur when the genetically-at-risk mice are housed in a conventional environment or infected with various specific bacteria. Exposure to probiotics or antibiotics also modifies the pattern of gut inflammation in these models of IBD.

In the setting of individuals with CD, a defunctioning surgical procedure (meaning that a segment of gut has no faecal flow with a consequent reduction in organisms) is typically followed by resolution of inflammation in the bypassed section of gut [22]. Inflammation, however, recurs soon on reversal of the defunctioning procedure, recommencement of flow and resumption of direct bacterial interaction with the mucosa [23]. Similarly, a prolonged period of antibiotics may delay the endoscopic recurrence of disease following a surgical resection [24].

## 4. Alterations in the intestinal microbiota secondary to EEN

Although EEN has been utilised to induce remission in individuals with active CD for many years, it has only been in the last few years that investigators have worked to elucidate the mechanisms by which this intervention leads to reduced inflammation. Several mechanisms have been demonstrated and proposed. These include enhanced barrier function with increased tight junction performance and direct anti-inflammatory effects during EEN therapy [25]–[27]. A number of reports have also clearly demonstrated that EEN leads to significant and relevant alterations in the intestinal microbiota.

The first reports that considered the direct effects of EEN on the intestinal microbiota utilised temperature temporal or density gradient gel electrophoresis (TTGE or DGGE respectively) methods. These reports demonstrated significant alterations consequent to the nutritional intervention [28]–[30]. For example, in an early study utilising the DGGE technique, the changes in the patterns of microbiota were clearly shown and persisted for some time but were not consistent across each of the included subjects [30]. Interestingly, the percentage similarity of Bacteroides-Prevotella group bacteria between week 0 and week 8 of EEN was positively correlated with the reduction in disease activity (utilising the Pediatric Crohn disease activity index: PCDAI) over this period. Later, in the period between 8 and 26 weeks after starting EEN, percent similarity of *Clostridium leptum* group was associated with change in disease activity and change in levels of S100A12 (a faecal marker of inflammation).

In a more recent report that also utilised TTGE (augmented with quantitative PCR), Gerasimidis et al. [31] characterised the effects of EEN in 15 children with CD along with paired samples from children without IBD. EEN led to a reduction in bacterial diversity, reflected by a narrow range of bacterial species present, compared to that seen in the paired samples from the control children. In the addition, the paired samples from the control children showed greater similarity over time than the paired samples from the children with CD (reflecting a loss of stability consequent to the nutritional intervention in the children with CD). Furthermore, Bacteroidetes concentrations were reduced during EEN, with subsequent recovery on resumption of normal diet. This report also demonstrated that 30 days of EEN resulted in reduced concentrations of *F. prausnitzii* species, contrary to the authors' hypothesis and contrary to expectations arising from the earlier observations made in adult patients with CD [19],[20].

As well as assessing the impact of EEN on the structure of the intestinal microbiota, these authors also characterised functional aspects subsequent to the period of nutritional intervention [31]. EEN was shown to result in a reduction in butyrate, a critical short chain fatty acid, along with an increase in faecal pH and levels of sulfides. These findings provide further information about the functional implications of the effects that EEN has on the microbiota. Although these changes may simply reflect preferential growth of sulfide producers, and suppressed growth of butyrate-producers, the reduction in butyrate may indicate a consequent reduction in colonocyte health during this time. This may indicate a therapeutic opportunity for the addition of butyrate along with EEN as an adjunctive intervention in order to more optimally support colonic function.

A number of subsequent studies have employed 16S rRNA high throughput sequencing and some reports have also employed whole genome or shot-gun sequencing techniques. Some additional reports have also utilised methods to further describe the functional implications of the changes in the microbiota.

One of the first studies to utilise 16S rRNA sequencing showed dysbiosis at the time of diagnosis of the five subjects with CD, which was not apparent in control children [32]. After commencing EEN, the number of operational taxonomic units (OTU) reduced quickly. This change correlated closely with the induction of remission. Later during the period of observation, an increase in the number of OTU was also associated with subsequent disease exacerbation. This report also indicated that the presence of specific Firmicutes strains were associated with disease activity during and after the period of EEN.

Quince et al. [33] evaluated the effects of EEN in 23 children with CD, with samples before, during and after EEN. Comparisons were made to samples collected from 21 healthy children. 16S sequencing and shot-gut metagenomics were utilised to assess the impact of the nutritional intervention. Overall, these data again illustrated that microbial diversity was less in the children with CD prior to EEN than the matched controls. EEN resulted in further reduction of diversity with greater differences from the control setting. Similar to earlier data, these investigators showed an association between changes in the flora and faecal calprotectin levels (a marker of gut inflammation).

Lewis and colleagues [34] assessed a large group of patients who were treated with a polymeric formula to provide 90% of caloric intake (along with some solid food intake). The EEN protocol utilised in this group of children differed from other EEN protocols, in that the enteral formula did not provide all caloric intake. This study also included two groups of children treated with other interventions: one group received a biologic therapy and the second group received partial enteral nutrition (with approximately half of their caloric intake with an enteral formula and half as solid foods). Comparisons were also made to samples from another group of children without IBD. Relative to the control children, the children treated with EEN had significant changes in the diversity of the microbiota within seven days of the intervention. Interestingly, more marked changes were seen in the children who responded to enteral nutrition than in those who did not respond. The abundance of six specific genera altered over this time. These early changes seen following EEN were not seen in the other two treatment groups.

This study also delineated the patterns of the intestinal microbiota after 8 weeks of therapy and stratified according to response (defined as reduction in faecal calprotectin below 250 mcg/g) or non-response. Response in the groups treated with EEN or a biologic therapy was associated with the microbiota reverting closer to the patterns seen in the control children.

More recently, Schwert and colleagues [35] utilised high throughput sequencing to characterise changes in the microbiota in conjunction with detailed immunological assays. Fifteen children with active CD were included, with stool samples for characterisation of the microbiota taken prior to and then after 2 weeks and 6–8 weeks of EEN. These analyses were correlated with assessments of disease activity and with immunological responses at baseline and after 3 weeks of EEN. As previously, EEN resulted in reductions in key groups of bacteria, namely those belonging to the *Bacteroidetes* phylum, and with increases in bacteria in the *Firmicutes* phylum. These changes were evident after just two weeks of EEN. Interestingly the changes in the children who were recently diagnosed differed from those with longstanding disease. In contrast to the findings described by Kaakoush et al. [32], these authors did not demonstrate reductions in the numbers of OTU during EEN therapy.

This study also characterised specific and detailed changes in innate and acquired immune status [35]. Specifically, EEN therapy was associated with a decreased number of T regulatory (Treg) cells in the lamina propria of the intestinal epithelium. The authors modelled network relationships between the altered microbiota and the immunological improvements during this short term dietary change. However, no analyses were conducted beyond the period of EEN and the timing of the different assessing modalities differed also.

Dunn et al. [36], in an assessment of the impact of EEN in ten children with IBD and five healthy controls, again showed that diversity was lower in individuals with CD compared to control subjects. Interesting, the data from this report demonstrated that diversity was lowest in those who proceeded to have a subsequent sustained remission. There were also differences in the structure of the microbiota in the children who remained in remission up to 24 weeks post diagnosis compared to those who did not. Those who remained well had a number of particular strains present, including Bacteroides, along with limitation in Proteobacteria. Those who had early relapse had a greater proportion of Proteobacteria. These differences were present at baseline: utilisation of these differences was then able to predict response rate with 80% accuracy.

Each of the studies reported above were conducted in children. To add to this pattern, two studies have been reported to date that included adult subjects. Jia et al. [37] assessed the impact of two weeks of an elemental formula (given exclusively) in adults with CD (in comparison to subjects with irritable bowel syndrome, UC and healthy controls) with PCR to characterise changes in *F. prausnitzii* alone. EEN was noted to reduce several types of this bacterium, similar to and supporting the changes described in the afore-mentioned paediatric study [31]. These data indicate that the administration of EEN appears to have particular effects on this group of bacteria.

Shiga and co-authors [38] assessed the impact of EEN in 30 adults with CD, using terminal restriction fragment length polymorphism methods augmented with quantitative PCR. An elemental formula was utilised over an average of 38 days in this study. This study showed similar findings to the paediatric reports that employed more recent molecular methodologies, with reductions in bacteria overall (reduced bacterial diversity). During the course of EEN in these patients the numbers of bacteria in the *Bacteroides fragilis* group reduced by one tenth: there were no significant changes in the other bacterial groups assessed.

The focus of the work reviewed above was on the impact of the EEN on the intestinal microbiota in the context of CD. Interestingly, there have been a series of reports demonstrating that this nutritional intervention is also efficacious and also leads to alteration of the structure and function of the intestinal microbiota in rheumatological disease. Swedish authors reported that a course of EEN given over 3 to 8 weeks in 13 children with juvenile idiopathic arthritis (JIA) promptly resulted in marked reduction of joint inflammatory scores and decreased levels of key pro-inflammatory cytokines implicated in joint inflammation [39]. This team have also assessed the effects of two separate, successive courses of EEN on the intestinal microbiota in one child with JIA [40]. EEN resulted in marked changes of the microbiota with particular reduction in the Bacteroidetes phylum. Furthermore, short chain fatty acid levels were decreased at the end of each course of EEN, with subsequent increases. The changes in the structure and function of the intestinal microbiota paralleled the observed clinical improvements. Although this work is preliminary (a single patient studied in detail), the data arising is promising, and indicates similar effects of EEN in the two disease states and hints at common mechanisms of action of EEN in children. Further work in both areas is clearly required to fully define the roles of the intervention and potentially to more fully elucidate the mechanisms of action.

## 5. Conclusions

There are now a number of reports that have utilised various techniques to elucidate the specific changes in the intestinal microbiota following the intervention of EEN in the setting of CD. Additional reports have also characterised some functional implications of the changes in the microbiota.

Overall, these reports have all shown that EEN leads to profound alterations in the intestinal microbiota, with these changes being rapid (within one week) and generally maintained throughout the dietary intervention. The reports have also emphasised significant interindividual variations. Interestingly, at least two reports have specifically demonstrated changes in *F. prausnitzii*, which has been characterised as a defensive bacterium in adults with CD. Whilst this reduction may just reflect the change in nutrient intake for this organism, it is intriguing that this reduction is concurrent with improved inflammation and disease activity, which is contrary to the previously-reported activity of this bacterium.

The changes in the abundance of *F. prausnitzii*, as noted in several reports, may reflect the change in nutrient supply for this bacterium. None of the enteral nutrition formulae utilised in the recent EEN studies contain dietary fibre. The lack of this may contribute to the consequent reduction in abundance of organisms that have fermentative activities. Consistent with this are the studies that have measured specific short chain fatty acids: each of these reports have indicated a reduction in levels of faecal butyrate, a product of fermentation of dietary fibre. This feature was also shown in the studies involving children with joint disease (without known gut inflammation) reflecting that the response is more likely secondary to the nutritional change.

Several reports have also demonstrated that the changes in the structure in the intestinal microbiota may be reflective of the duration of disease (newly-diagnosed versus long-standing disease) and also provide predictive pointers to the response to EEN itself. Other reports have demonstrated that the changes seen in response to EEN are subsequently reversed in those who have an exacerbation of their disease. Together these findings indicate that characterisation of the structure of the intestinal microbiota may have significant prognostic relevance in children with CD. Prompt assessments of key aspects of the intestinal microbiota may be able to be utilised to predict the response to EEN, and also to predict the risk of subsequent relapse of disease. Inclusion of these bio-indicators in a panel with other specific bio-markers (e.g. inflammatory markers such as faecal calprotectin) may be even more advantageous.

At present the reports of the impact of EEN on the intestinal microbiota do not fully illuminate the mode of action of this intervention in the context of active CD. The network modelling of Schwert et al. [35] suggest that changes in the flora may interact directly with the intestinal epithelium and local/systemic immune responses. However, these immunological events may result from direct effects of the enteral formulae utilised for EEN on the epithelium, rather than be mediated through changes in the microbiota. Other work has demonstrated that these same polymeric formulae have direct anti-inflammatory and protective effects on the epithelium [25]–[27]. The interactions between these mucosal changes and the microbiota have not yet been characterised. As a result, it is not yet clear if the modifications in the intestinal microbiota seen after EEN are simply a direct consequence of altered dietary intake or if the changes in the microbiota are a direct effect that then also modulates the mucosal events. Further work to understand these complex interactions is required: as well as enabling optimisation of EEN protocols, such advances in understanding should also provide clues about the pathogenesis of CD.

Although the data outlined in this report have focused on individuals with CD, it is intriguing to note that EEN has also been demonstrated to have benefits in other inflammatory conditions, such as joint disease. Combined studies of dietary interventions in various inflammatory conditions may also help to further advance the roles and mechanisms of EEN.

In conclusion, there is no doubt of the role of the intestinal microbiota in the development and pathogenesis of IBD, especially CD. These data demonstrate that nutritional intervention in individuals with CD is associated with wide and profound changes in the patterns of the microbiota. These data give strong indications that these changes may be employed in prognostic or predictive models to enhance the outcomes of individuals with CD.
